# Effects of Ketoprofen and Morphine on Pain-Related Depression of Nestlet Shredding in Male and Female Mice

**DOI:** 10.3389/fpain.2021.673940

**Published:** 2021-06-08

**Authors:** Jamani B. Garner, Laura S. Marshall, Nathaniel M. Boyer, Vinaya Alapatt, Laurence L. Miller

**Affiliations:** Department of Psychological Sciences, Augusta University, Augusta, GA, United States

**Keywords:** pain, behavior, impairment, depression, ketoprofen, morphine, nesting

## Abstract

A primary goal in pain treatment is restoration of behaviors that are disrupted by pain. Measures of pain interference indicate the degree to which pain interferes with activities in pain patients, and these measures are used to evaluate the effects of analgesic drugs. As a result of the emphasis on the expression and treatment of functional impairment in clinical settings, preclinical pain researchers have attempted to develop procedures for evaluation of pain-related functional impairment in laboratory animals. The goal of the present study was to develop and validate a low cost procedure for the objective evaluation of pain-related depression of home cage behavior in mice. On test days, a 5 × 5 cm Nestlet was weighed prior to being suspended from the wire lid of the home cage of individually housed male and female ICR mice. Over the course of experimental sessions, mice removed pieces of the suspended Nestlet, and began to build a nest with the material they removed. Thus, the weight of the pieces of Nestlet that remained suspended at various time points in the session provided an indicator of the rate of this behavior. The results indicate that Nestlet shredding was stable with repeated testing, and shredding was depressed by intra-peritoneal injection of 0.32% lactic acid. The non-steroidal anti-inflammatory drug ketoprofen blocked 0.32% lactic acid-induced depression of shredding, but did not block depression of shredding by a pharmacological stimulus, the kappa opioid receptor agonist U69,593. The μ-opioid receptor agonist morphine did not block 0.32% lactic acid-induced depression of shredding when tested up to doses that depressed shredding in the absence of lactic acid. When noxious stimulus intensity was reduced by decreasing the lactic acid concentration to 0.18%, morphine was effective at blocking pain-related depression of behavior. In summary, the data from the present study support consideration of the Nestlet shredding procedure for use in studies examining mechanisms, expression, and treatment of pain-related functional impairment.

## INTRODUCTION

Pain remains a significant public health issue with some estimates suggesting that close to 40% of adults experience some sort of chronic pain condition (1). Consistent with the prevalence of pain, research on the mechanisms and treatment of pain is a high priority. For instance National Institutes of Health (NIH) funding for research on pain and related issues has increased in recent years from ~$500 million in 2014 to an estimated $924 million in 2020 (2). Key goals for pain research include the development of novel treatments with clinically meaningful functional improvement (3). Bidirectional translational research represents one strategy for achieving this goal. This strategy indicates that basic research should inform clinical practice, and clinical questions and phenomena should inform basic research. Attempts to mimic clinically relevant pain states (e.g., neuropathic pain, inflammatory pain, disease state) in clinically relevant anatomical features in preclinical models is one example of clinical phenomena informing basic research. This example also reflects usage of the phrase “pain model” where emphasis is placed on the variables associated with the pain state (4). An alternative but complementary approach to using clinical phenomena to inform basic science, is the use of models of pain-related functional impairment (5). This approach reflects an acknowledgment of the importance of the dependent variable or target behavior that is used, and the potential value of “modeling” clinically relevant consequences of pain including disruption of behavior.

Recently, as part of a broader movement beyond focusing on reflexive behaviors that are stimulated by pain stimuli, researchers have developed models of pain-related functional impairment or pain-related depression of behavior. Consistent with pain-related depression of rates of behavior observed in humans and in veterinary medicine, commonly used preclinical research subjects such as mice and rats display pain-related depression of behavior. A number of studies have shown that pain stimuli commonly used in studies that focus on pain-related stimulation of reflexive behaviors [e.g., intra-peritoneal injection of dilute acid, paw incision, and intra-plantar injection of Complete Freund’s Adjuvant (CFA)] also depress behavior in rodents. Behaviors depressed by these manipulations include conditioned behaviors such as operant responding for food and other reinforcers (6–8), and unconditioned behaviors such as feeding, wheel running, burrowing, and nesting (9–13). Such findings and their parallels with pain-related depression of behavior in human and veterinary patients provide evidence of the face validity of this approach to studying pain. Studies showing that clinically effective analgesics such as non-steroidal anti-inflammatory drugs (NSAIDs) and opioids block pain-related depression of behavior provide further evidence in supporting the use of this approach to complement assessment of pain-stimulated behavior (14, 15).

The purpose of the present study was to develop and assess the validity of a simple, low cost, procedure for examining pain-related depression of behavior in mice. This study builds on previous research showing that mouse behaviors related to nesting are sensitive to depression by pain manipulations (13, 15–19). Nesting behaviors are an attractive target behavior in mouse studies of pain-related depression of behavior because they are innate (i.e., do not require training), can be completed with minimal costs beyond basic animal care costs, and can potentially be completed as part a relatively rapid and unobtrusive assessment. The target behavior in the current study was shredding and removal of nesting material that had been suspended from the wire top of the home cage. In addition to incorporating the innate tendency of mice to build nests, this target behavior incorporates rearing, which is another behavior that is sensitive to depression by pain stimuli (20). The target behavior was quantified by measuring the percent of material removed. Initial experiments determined the baseline rate of the behavior in the absence of any manipulation. Next, the effects of the visceral noxious stimulus intra-peritoneal injection of lactic acid (IP acid) were determined. Finally, experiments were conducted to determine the ability of ketoprofen and morphine to block lactic acid induced depression of shredding, and to determine the selectivity of these effects, as applicable. We predicted that lactic acid would depress shredding, and that both ketoprofen and morphine would block this effect.

## METHODS

### Subjects

Subjects were 57 adult male (*N* = 30) and female (*N* = 27) ICR mice that were 10 weeks old and weighed 23–45 g upon arrival in the laboratory. Details on the number of mice per group for each experiment is indicated in the associated figure captions. Mice were housed individually in plastic cages (27.9 cm long × 16.5 cm wide × 12.7 cm deep) supplied with corncob bedding, one 5 × 5 cm “Nestlet” (Ancare, Bellmore, NY) composed of pressed virgin cotton, and *ad libitum* access to food (Teklad 2918) and water. Cages were placed on a rack in a temperature-controlled room (23–24°C). Lights in the room were maintained on a 12-h light/dark cycle with lights off from 6:00 a.m. to 6:00 p.m. Testing was conducted during the dark phase (8:00 a.m.–2:00 p.m.) and was initiated no sooner than 48 h after arrival from the vendor. All experiments below were conducted with a repeated measures design. Mice were euthanized by CO2 exposure followed by decapitation after completion of the procedures described below. Animal use protocols were approved by the Augusta University Institutional Care and Use Committee, and comply with the National Research Council Guide for the Care and Use of Laboratory Animals (21).

### Shredding Procedure

Shredding behavior was initially assessed during 120-min sessions conducted to characterize the behavior in the absence of pain or pharmacological stimuli. Prior to the start of shredding sessions, each 5 × 5 cm Nestlet used in the study was weighed. At the start of the sessions, mice were temporarily removed from the home cage while existing nesting material was removed and a new 5 × 5 cm Nestlet was suspended from the wire lid of the cage using a binder clip (Staples, 3002, 0.75”W). Mice were then returned to the cage, the session started (0 min), and the experimenter left the room. Subsequently, the experimenter returned at various intervals after the start time (10, 30, 60, 120, 150, and 180 min) to weigh the portion of the Nestlet that remained suspended from the lid of the cage by the binder clip. Our initial observations revealed that during these intervals the mice would approach the suspended Nestlet, rear up on the hind limbs, and shred and pull material away from the suspended Nestlet. This behavior was quantified at each test interval by calculating the percent of material removed at each time point (% Removed) using the equation [(0 min weight-test interval weight)/0 min weight]*100. Following initial characterization of the behavior under control conditions, the 30 min time point was selected for experiments examining the effects of intra-peritoneal injection of dilute lactic acid, and test drugs.

### Noxious Stimuli and Pharmacological Treatments

Mice were randomly assigned to groups of 10–12 animals to examine the effects of IP acid, ketoprofen, morphine, and the kappa opioid agonist, U69,593 (see results for details on the number of males and females per group). All of these experiments were conducted using a repeated measures design with at least 72 h between test sessions. First, the effects of IP acid were examined by administering an intra-peritoneal injection saline 30 min prior to the start of the session (−30 min) followed by a range of concentrations of lactic acid (0.1–0.32%; 0.25-log increments) immediately before the start of the session (0 min). The order of lactic acid concentration exposure for each mouse was determined by Latin-square. Based on these experiments, 0.32% lactic acid was used for initial experiments examining drug effects. Next, the effects of ketoprofen were examined (0.01–1.0 mg/kg; 0.5-log increments). In these experiments, one dose of ketoprofen was tested twice each week, and the order of exposure was determined by Latin-square. During these tests, ketoprofen was administered 30 min prior to administration of IP acid or acid vehicle at the 0 min time point. The order IP acid/acid vehicle exposure during these tests was counterbalanced across mice. Because ketoprofen was effective at preventing IP acid-induced depression of shredding (see results), experiments were then conducted with U69,593 to examine the selectivity of ketoprofen’s effects. First we tested a range of doses of U69,593 (0.1–3.2 mg/kg; 0.5-log increments) to identify a dose that produced robust depression of shredding. We then used this dose (1.0 mg/kg) to examine ketoprofen’s (0.1–1.0 mg/kg; 0.5-log increments) ability to block depression of shredding by a pharmacological stimulus. Finally, the effects of morphine (0.1–10 mg/kg; 0.5-log increments) were examined. These experiments were conducted in a manner similar to ketoprofen, with morphine administered 30 min prior to administration of IP acid or acid vehicle at the start of the session. Morphine data were initially collected with 0.32% lactic acid as discussed above, and follow up experiments determined morphine effects with a lower magnitude noxious stimulus (0.18%).

### Drugs and Noxious Stimulus

Lactic acid (Sigma-Aldrich, St Louis, MO) was diluted in sterile water, and ketoprofen (Sigma-Aldrich, St Louis, MO) was dissolved in a solution of sterile water, ethanol, and kolliphor (92.5, 5, 2.5%; Sigma-Aldrich, St Louis, MO). Lactic acid and ketoprofen were administered by intraperitoneal injection with a 27 G needle, at a volume of 10 ml/kg. Morphine (Sigma-Aldrich, St Louis, MO) was dissolved in saline and administered subcutaneously with a 27 G needle, at a volume of 10 ml/kg. U69,593 (Sigma-Aldrich, St Louis, MO) was dissolved in a small amount of lactic acid and then diluted with saline to a final concentration of 0.056% lactic acid.

### Data Analysis

For each acid concentration or drug dose, data for % Removed (see above) was averaged across mice, and evaluated by one-way or two-way repeated measures analysis of variance (ANOVA), followed by Dunnett’s or Sidak’s multiple comparisons when appropriate. The criteria for significance was *P* < 0.05 for all analyses.

## RESULTS

### Baseline Shredding

**FIGURE 1 |:**
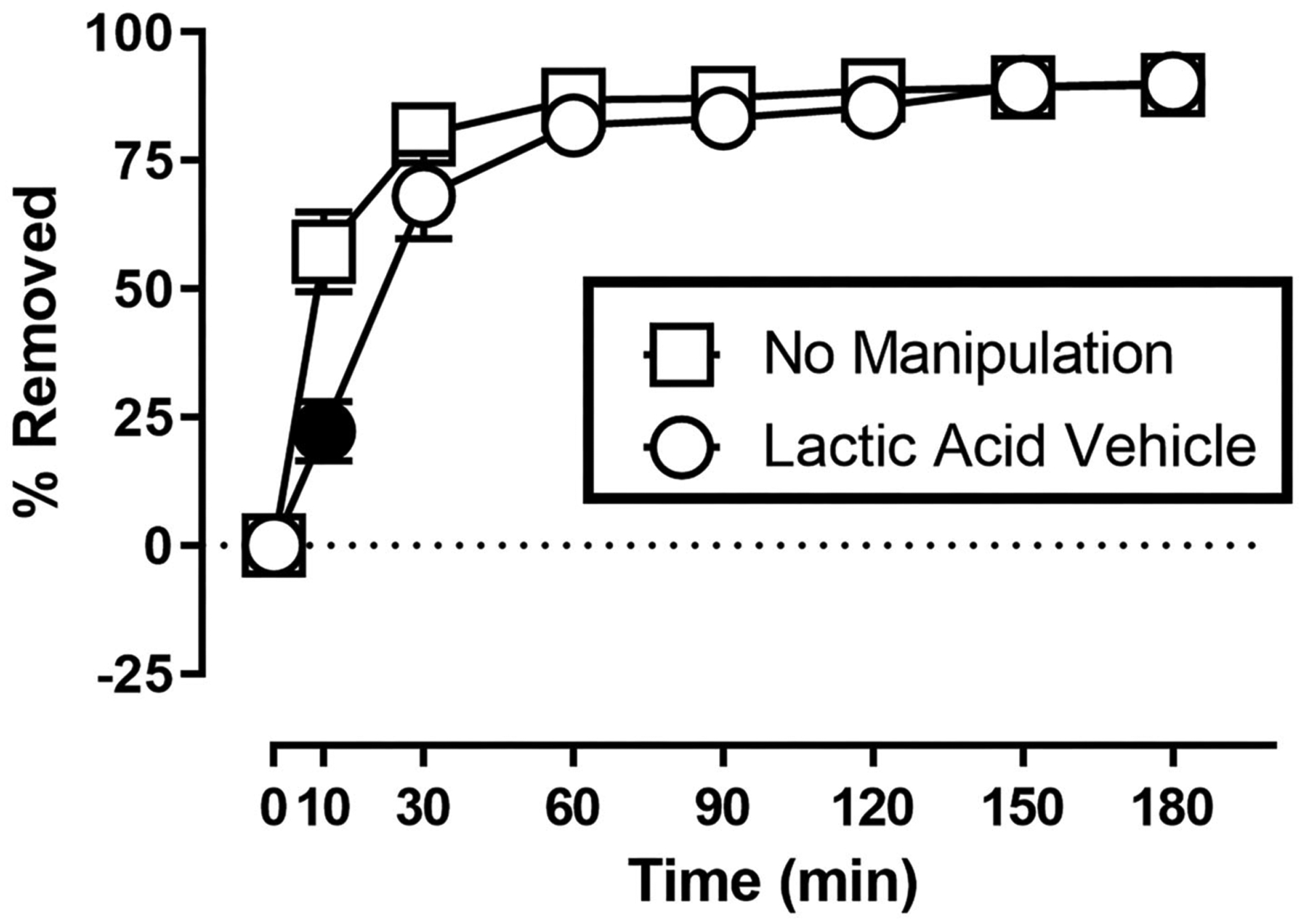
Suspended Nestlet shredding under baseline and control conditions. The abscissa shows session time, and the ordinate shows mean % Removed. “No Manipulation” (squares) indicates mice received no treatment prior to the session. “Lactic Acid Vehicle” (circles) indicates mice received an intraperitoneal injection of sterile water immediately prior to the session. Symbols show mean ± SEM, and represent data from 12 mice (seven males and five females). Filled symbols indicate a significant difference compared to the no manipulation condition at that time point.

Figure 1 shows the rate of Nestlet shredding in animals receiving no manipulation, and after receiving an injection of sterile water (lactic acid vehicle) immediately before the start of the session. Two-way ANOVA indicates significant effects of Time [*F*_(1.608,17.68)_ = 147.6; *p* < 0.05] and Treatment [*F*_(1.000,11.00)_ = 8.230; *p* < 0.05], and a significant Time X Treatment interaction [*F*_(2.353,25.89)_ = 12.31; *p* < 0.05]. Mice in both conditions removed the majority (> 68%) of nesting material within the first 30 min of the session, but Sidak’s multiple comparisons test indicates intraperitoneal injection of sterile water decreased % Removed at the 10 min time point.

### Effects of Lactic Acid

**FIGURE 2 |:**
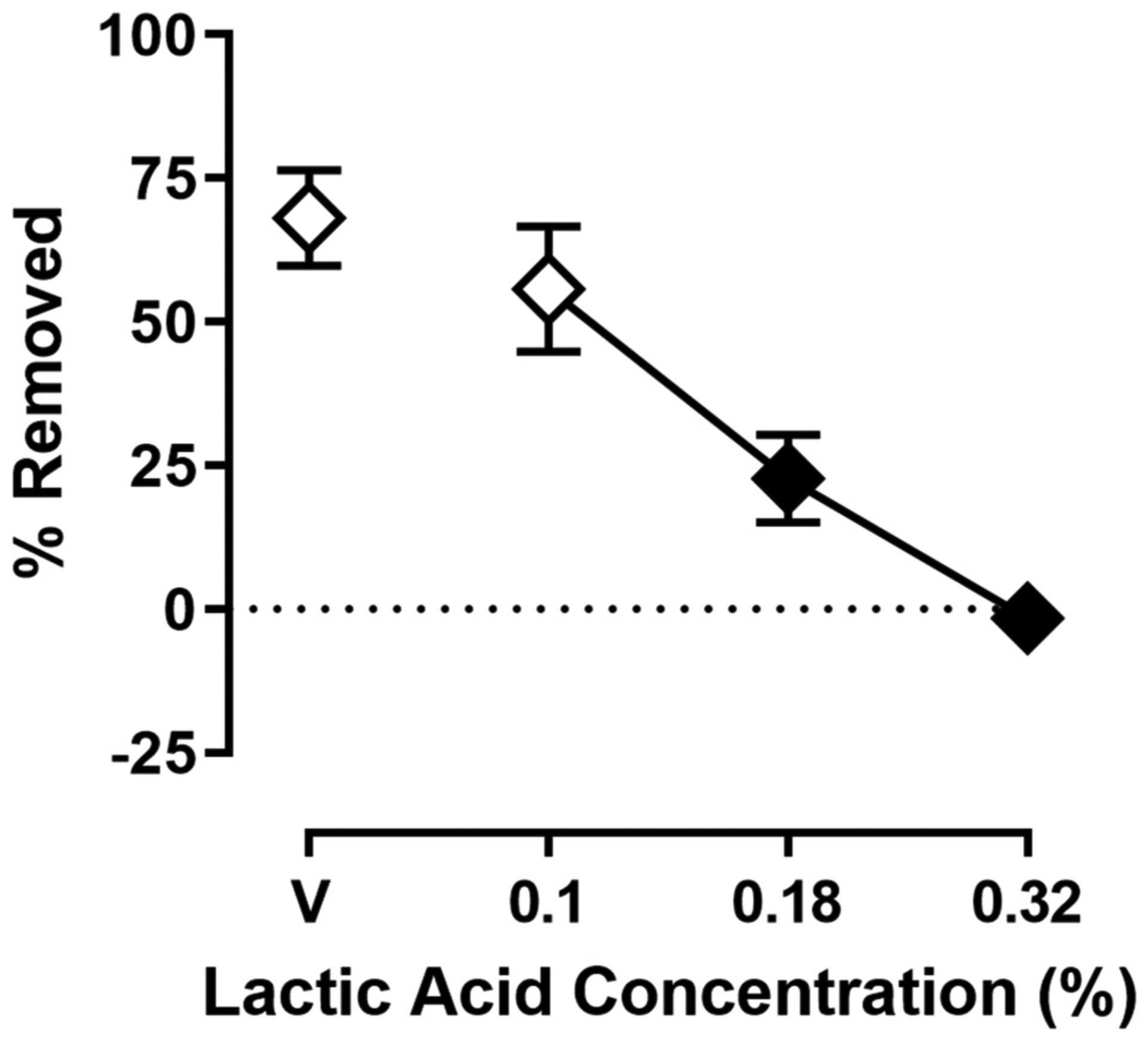
Effects of intraperitoneal lactic acid on Nestlet shredding. The abscissa shows lactic acid concentration, and the ordinate shows mean % Removed. Symbols show mean ± SEM, and represent data from 12 mice (seven males and five females). The same mice used for experiments associated with Figure 1 were used in experiments associated with this figure. Filled symbols indicate a significant difference compared to lactic acid vehicle.

Figure 2 shows the effect of lactic acid on % Removed at the 30 min time point of the session. One-way ANOVA indicates that lactic acid produced concentration-dependent depression of shredding [*F*_(2.240,24.64)_ = 19.88; *p* < 0.05], and Dunnett’s multiple comparison’s test indicates shredding is significantly depressed by 0.18 and 0.32% compared to lactic acid vehicle. The 0.32% concentration of lactic acid was selected for use in drug experiments based on the robust depression of shredding produced by this stimulus.

### Effects of Ketoprofen on Acid-Induced Depression of Shredding

**FIGURE 3 |:**
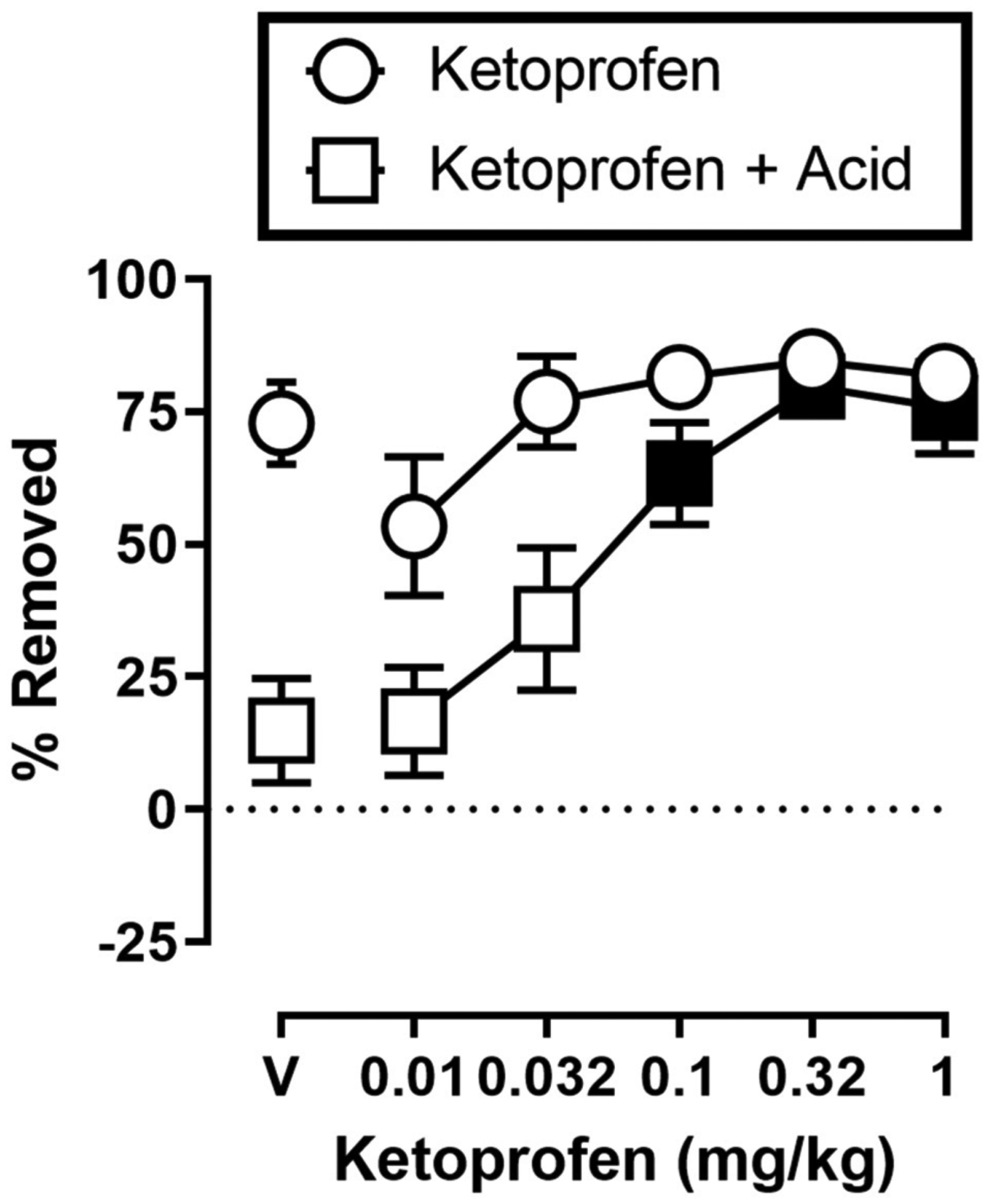
Effects of ketoprofen on IP acid-induced depression of Nestlet shredding. The abscissa shows ketoprofen dose, and the ordinate shows mean % Removed. Circles indicate data for ketoprofen alone, and squares indicate data for ketoprofen administered prior to IP acid. Symbols show mean ± SEM, and represent data from 12 mice (five males and five females). Filled points indicate a significant difference compared to lactic acid in the absence of ketoprofen.

Figure 3 shows the effects of ketoprofen on IP acid induced depression of shredding, and effects of ketoprofen alone at the 30 min time point of the session. One-way ANOVA indicates that ketoprofen dose-dependently blocked acid-induced depression of shredding [*F*_(3.486,31.37)_ = 11.15; *p* < 0.05], and Dunnett’s multiple comparisons test indicates that 0.1, 0.32, and 1.0 mg/kg significantly blocked acid-induced depression of shredding. Ketoprofen did not significantly affect shredding when administered in the absence of IP acid [*F*_(2.405,21.64)_ = 2.925; *p* > 0.05].

### Effects of Ketoprofen on U69,593-Induced Depression of Shredding

**FIGURE 4 |:**
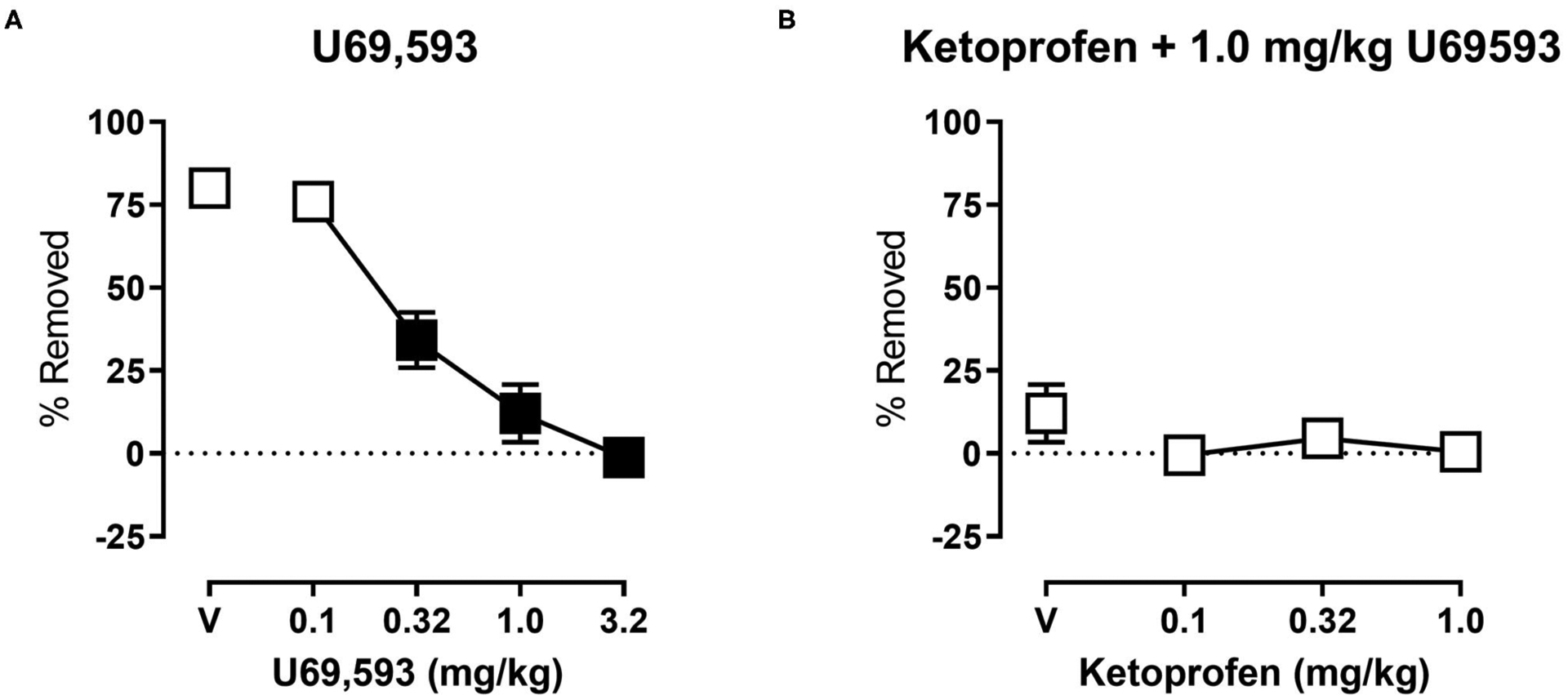
**(A)** Effects of U69,593 on Nestlet shredding. The abscissa shows U69,593 dose, and the ordinate shows mean % Removed. **(B)** Effects of ketoprofen on 1.0 mg/kg U69,593-induced depression of Nestlet shredding. The abscissa shows ketoprofen dose, and the ordinate shows mean % Removed. Symbols show mean ± SEM, and represent data from 12 mice (six males and five females). Filled points indicate a significant difference compared to vehicle.

Figure 4A shows the effects of U69,593 on shredding at the 30 min time point of the session. One-way ANOVA indicates that U69,593 produced dose-dependent depression of shredding [*F*_(2.643,26.43)_ = 41.18; *p* < 0.05], and Dunnett’s multiple comparisons test indicates that 0.32, 1.0, and 3.2 mg/kg significantly depressed shredding. The 1.0 mg/kg dose of U69,593 was selected to examine the ability of ketoprofen to block depression of shredding by a pharmacological stimulus. Figure 4B shows effects of ketoprofen on U69,593-induced depression of shredding. One-way ANOVA indicates that ketoprofen had no effect on depression of shredding by U69,593 [*F*_(1.271,12.71)_ = 1.282; *p* > 0.05].

### Effects of Morphine on Acid-Induced Depression of Shredding

**FIGURE 5 |:**
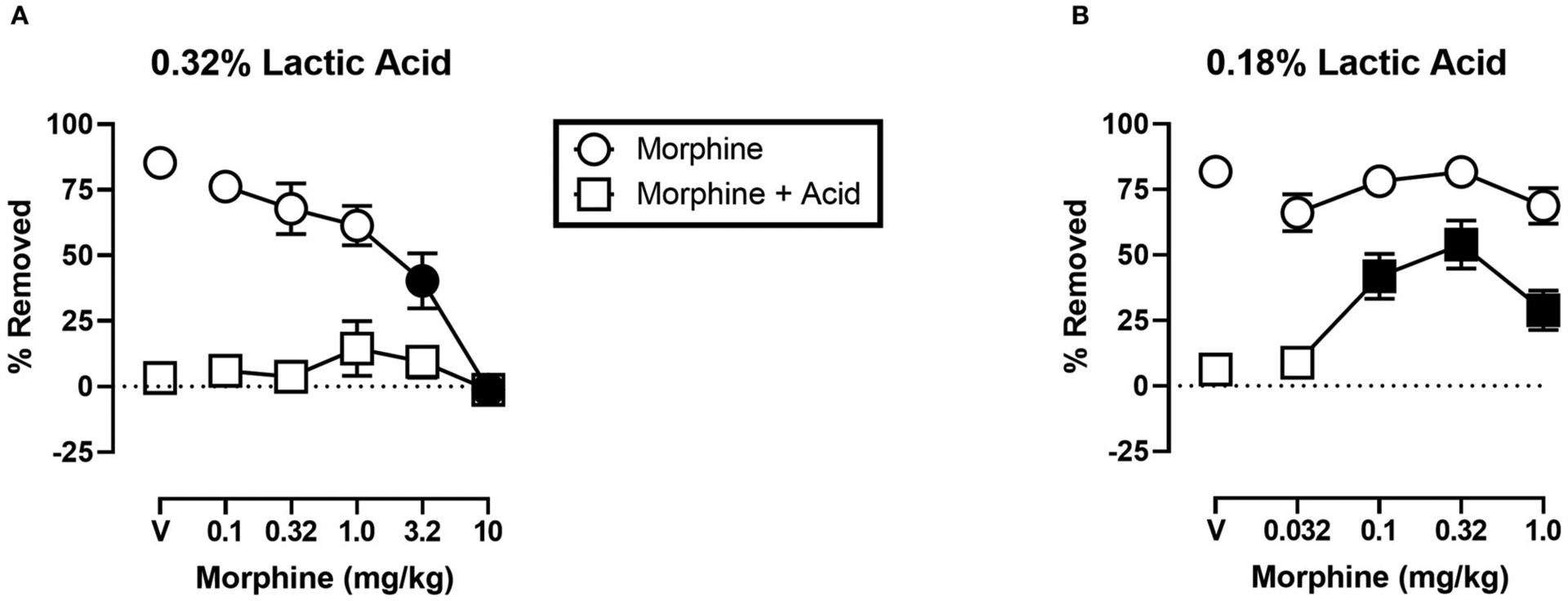
**(A)** Effects of morphine on 0.32% IP acid-induced depression of Nestlet shredding. **(B)** Effects of morphine on 0.18% IP acid-induced depression of Nestlet shredding. The abscissa shows morphine dose, and the ordinate shows mean % Removed. Circles indicate data for morphine alone, and squares indicate data for morphine administered prior to IP acid. Symbols show mean ± SEM, and represent data from 12 mice (six males and five females). Separate groups of mice were used for the experiments associated with each panel. Filled points indicate a significant difference compared to morphine vehicle.

Figure 5 shows the effects of morphine on IP acid-induced depression of shredding, and effects of morphine alone at the 30 min time point of the session. Experiments were conducted to examine morphine effects on 0.32% lactic acid (Figure 5A), and 0.18% lactic acid (Figure 5B). One-way ANOVA indicates that morphine had no effect on depression of shredding by 0.32% IP acid [*F*_(2.122,23.35)_ = 0.9453; *p* > 0.05]. One-way ANOVA indicates that morphine did dose-dependently depress shredding when administered in the absence of lactic acid [*F*_(2.945,32.40)_ = 21.84; *p* < 0.05]. Dunnett’s multiple comparisons test indicates that 3.2 and 10.0 mg/kg significantly depressed shredding. One-way ANOVA indicates that morphine significantly blocked depression of shredding by 0.18% IP acid [*F*_(2.176,23.94)_ = 10.34; *p* < 0.05]. Dunnett’s multiple comparisons test indicates that 0.1, 0.32, and 1.0 mg/kg morphine blocked acid-induced depression of shredding. One-way ANOVA indicates that the lower range of doses tested in this experiment (Panel B) had no effect on shredding when administered in the absence of lactic acid [*F*_(1.887,20.75)_ = 2.507; *p* > 0.05].

## DISCUSSION

The purpose of the present study was to develop and assess the validity of a simple, low cost, procedure for examining pain-related depression of behavior in mice. The suspended Nestlet shredding procedure can be conducted with standard mouse housing supplies, and inexpensive office supplies such as binder clips. Thus, the costs associated with the procedure are minimal. The procedure produces baseline data that is stable within subjects, and as a result a repeated measures design is feasible if desired. Intra-peritoneal injection of dilute acid, a pain stimulus capable of both stimulating behavior (22) and depressing behavior (23) was effective at depressing Nestlet shredding. Results with established analgesics support the validity of this procedure as a tool for studying pain-related functional impairment, and its treatment by candidate analgesics.

Increasingly, preclinical pain researchers are working to develop methods for the study of pain that move beyond conventional measures of pain-stimulated behavior. Assays of pain-stimulated behavior rely on reflexive unconditioned behaviors (24) and include withdrawal responses and pseudoescape responses following exposure to pain stimuli. The hotplate and writhing procedures are representative of this approach. Although these procedures are sensitive to analgesic drug effects, they have limitations that have led to the push for consideration of new approaches. These limitations and strategies to overcome them have been discussed in detail elsewhere (4, 24), but one consideration is that these procedures do not address at least one key clinically relevant consequence of pain: pain-related functional impairment. In response to this issue, researchers have developed procedures with pain-related functional impairment in mind.

One approach to studying pain-related functional impairment is the use of operant procedures in which a pain stimulus serves as a contextual stimulus (24). Here the experimental subject learns to perform a response such as a lever press that results in the delivery of a reinforcer such as food or electrical brain stimulation, and rates of behavior can be compared in the presence and absence of the pain stimulus. Studies using this approach have shown that operant behavior is sensitive to depression by pain stimuli in an analgesic reversible manner (6, 8, 25–28). Operant procedures have features that facilitate understanding and control of baseline rates of behavior, which could promote improved understanding of the expression, mechanisms, and treatment of pain-related functional impairment. Drawbacks to the use of operant procedures include the need for potentially expensive equipment, and extensive training of animals.

An alternative approach to studying pain-related functional impairment focuses on unconditioned responses to unconditioned stimuli where pain stimuli serve as a contextual stimulus (24). Here, the experimental subject engages in a behavior that is elicited by presentation of a stimulus, and rates of this behavior can be compared in the presence and absence of a pain stimulus. Examples of this approach include pain-related depression of feeding (23, 26), wheel-running (9, 10, 12, 29, 30), burrowing (11, 31–34) and nesting. Some previous studies have used rating systems to indicate nest quality (32), but recent studies use variations of a general approach that involves assessment of the collection and consolidation of nesting material within the home cage which potentially allows for more objective measurement of the dependent variable (13–19, 35).

One nesting procedure that we have used to examine the expression and treatment of pain-related depression of behavior involves distribution of nesting material across six “zones” of the home cage at the beginning of the session, and subsequent assessment of the number of zones cleared of material as the mouse consolidates the material into one zone while building the nest (13, 17). In this procedure the maximum score is five zones cleared which reflects the consolidation of the nesting material into one of the six zones. The minimum score is zero zones cleared which potentially reflects that the mouse has not moved the nesting material. Findings that pain stimuli depress zones cleared in an analgesic reversible manner provide support for the validity of this approach. This approach is also relatively inexpensive, and easily conducted. However, continued experience with this procedure revealed limitations that contributed to the decision to develop the current procedure. For instance, low scores in the “zones cleared” procedure can result from the mouse not consolidating the material (e.g., as a result of pain), but a low score can also result from the mouse consolidating the material in a location that overlaps multiple zones. Specifically, we have observed untreated animals that consolidate material and build what might be rated as a high quality nest, but that animal would receive a low zones cleared score because the nest overlapped four zones of the cage. Another observation from continued work with this procedure is that it is possible for mice to consolidate material into a compact area of the cage (i.e., a high zones cleared score), only to redistribute the material back into the other zones of the cage at a later time point. These two examples highlight the potential influence of extraneous variables on data from procedures that rely on nesting material consolidation.

The current procedure was developed with these issues in mind. The target behavior in this procedure (shredding) is a precursor to nest building, and there is no direct assessment of nest building. As a result, the data generated by this procedure is not sensitive to the location of the nest that is built with the shredded material. The procedure also addresses the possibility that a mouse can redistribute nesting material after previously consolidating it. In the current procedure, once the mouse removes the material from the suspended Nestlet, it cannot be replaced. Thus, the current procedure addressed some limitations of procedures that rely on nesting material consolidation, but shares some advantageous features of those procedures. These features include reliance on an innate behavior that occurs in the home cage and does not require much additional equipment or supplies beyond basic animal care supplies, limited direct interaction between the mouse and experimenter once the experimental session starts, and generation of quantitative data on a ratio scale that is appropriate for parametric statistics.

At face value, the current procedure has many appealing features as discussed above, however pharmacological validation is important in determining how useful a procedure will be for the development and assessment of candidate analgesics. In the current study we used two established analgesics to assess the validity of this procedure. Consistent with previous studies using a variety of assays of pain stimulated behavior (13, 17, 26, 36), we found that the NSAID ketoprofen dose-dependently blocked pain-related depression of shredding. Moreover, the present study showed that ketoprofen’s effects were selective for blocking pain-related depression by intraperitoneal injection of lactic acid, and did not impact depression of behavior by a pharmacological stimulus, the kappa opioid agonist U69,593. Together, these findings support the validity of this procedure for examination of pain-related depression of behavior in mice.

Results with morphine were more complex. Morphine did not block depression of shredding by 0.32% lactic acid. Previous research showed morphine-induced anti-nociception using a different nesting-related behavior, ICR mice, and the same concentration of lactic acid (17). The fact that we tested doses ranging from those without an effect on control behavior to those that significantly depressed control behavior suggested that we tested a relevant range of doses. These results did not support the validity of this procedure for examination of pain-related depression of behavior in mice, and led us to consider why we obtained these unexpected result.

One observation is that, unlike ketoprofen, morphine disrupted shredding when administered in the absence of lactic acid. These effects were dose-dependent with 3.2 and 10.0 mg/kg morphine producing significant depression of shredding, and 1.0 mg/kg morphine producing a non-significant decrease in shredding. As can be seen in Figure 5A, morphine administration prior to IP acid produced a flat inverted U-shaped dose-effect relationship with 1.0 mg/kg morphine producing a non-significant increase in % Removed. Thus, it is possible that decreases in shredding behavior produced by morphine limit the expression of anti-nociception as defined in this procedure. Clinical evidence indicates that tolerance to certain opioid side effects such as sedation develops relatively rapidly (37), and preclinical data suggests that tolerance to behaviorally disruptive effects can unmask or enhance anti-nociceptive effects in assays of pain-related depression of behavior (27). Though the current study used a repeated measures design that resulted in animals repeatedly being treated with morphine, this regimen would not be expected to result in tolerance and examination of the data are consistent with this conclusion. Thus, future studies that include manipulations that attenuate morphine-induced functional impairment might unmask anti-nociception in the shredding procedure.

Another method of altering opioid effects in pain procedures is by manipulating noxious stimulus intensity. Manipulation of thermal noxious stimulus intensity the most common example of this, probably due to the relative ease of manipulating temperature parameters compared to other pain manipulations. Increases in thermal noxious stimulus intensity are associated with decreases in opioid analgesic potency (38–41), and effectiveness (40, 42–47). Similar findings were obtained in an assay of lactic acid-induced depression of ICSS which showed decreased potency of opioid analgesics when lactic acid concentration was increased (41).

Based on these findings on the effects of noxious stimulus intensity, and our own findings with morphine and 0.32% lactic acid in the present study, we decided to examine the ability of morphine to block the depression of shredding by 0.18% lactic acid. Under these lower noxious stimulus intensity conditions morphine was effective at blocking lactic acid-induced depression of shredding. Thus, the present findings are consistent with previous studies showing a negative relationship between noxious stimulus intensity and the effectiveness of opioid analgesics. These data support the validity of this procedure for the assessment of candidate analgesics, and suggest that future studies examining interactions of noxious stimulus intensity and mu opioid agonist intrinsic efficacy will be useful in understanding the pharmacology of opioids in assays of pain-related depression of behavior.

The present data, along with morphine data from previous studies on pain-related depression of nesting behaviors (17) also suggest that the motor requirements of the target behavior might be a key determinant of drug effects in assays of pain-related depression of behavior. The shredding behavior from the present study requires mice to engage in rearing to reach the suspended Nestlet. Negus et al. (17) used a measure of nesting material consolidation that does not require rearing. Although the effects of lactic acid were qualitatively similar and concentration dependent in these two studies, 0.32% lactic acid essentially eliminated shredding in the present study, but did not completely eliminate nesting material consolidation in Negus et al. (17). One previous study examined the effect of raising and lowering lever height in an assay of pain-related depression of lever pressing (48). This manipulation of the response requirement impacted the baseline rate of lever pressing, but did not do so in a way that interacted with the effects of two pain stimuli: intra-plantar injection of complete Freund’s adjuvant and intra-plantar injection of formalin. Future studies would be required to further understand potential interactions between noxious stimulus intensity, drug effects, and other variables such as motor requirements in assays of pain-related depression of behavior.

In summary, this study evaluated an inexpensive and unobtrusive way to measure home cage behavior in mice, and provides data that support its consideration for use in studies examining mechanisms, expression, and treatment of pain-related functional impairment. The results with morphine indicate the importance of considering noxious stimulus intensity and perhaps other experimental features such as motor requirements as a determinant of drug effects in basic pain research and analgesic drug development. It will also be important to examine the impact of other pain manipulations on shredding behavior. Intra-peritoneal injection of lactic acid is an appealing noxious stimulus for reasons that include effects that are sensitive to established analgesics, the ability to manipulate noxious stimulus intensity, and the ability to use a repeated measures design. Studies examining other pain stimuli and manipulations that differ with regard to variables including mechanism and time course will be needed to understand the degree to which the present results can be generalized. Assessment of other variables including drug time course, sex, strain, and age would also contribute to better understanding of the potential for the procedure to contribute to the development of new knowledge on pain, and aid in analgesic drug development.
